# Associations between echocardiographic findings and prospective changes in residual renal function in patients new to peritoneal dialysis

**DOI:** 10.1038/s41598-019-54851-2

**Published:** 2019-12-05

**Authors:** Sara Mahdavi, Kibar Yared, George Wu, Billy Omar, Dinesh Savundra, Gordon Nagai, Edgar Hockmann, Anton Svendrovski, Antonio Bellasi, Paul Tam, Tabo Sikaneta

**Affiliations:** 1 Department of Nephrology, The Scarborough Hospital, Toronto, Canada; 2 Faculty of Family and Community Medicine, University of Toronto, Toronto, Canada; 3 Faculty of Medicine, University of Toronto, Toronto, Canada; 4 Department of Medicine, Trillium Health Partners, Mississauga, Canada; 5 Private Biostatistics Consultant, Toronto, Canada; 6 Aziende Socio Sanitarie Territoriale Papa Giovanni XXIII, Bergamo, Italy

**Keywords:** End-stage renal disease, Peritoneal dialysis

## Abstract

Although echocardiograms are often performed when peritoneal dialysis is started, associations between commonly reported findings and prospective changes in renal function remain understudied. Ninety-nine of 101 patients in the Trio Trial had transthoracic echocardiograms within 6 months of dialysis initiation, and measurements of residual renal function every six weeks for up to two years. Generalized mixed modelling linear regression in STATA was used to examine associations between left atrial size, left ventricular hypertrophy, left ventricular ejection fraction, right ventricular systolic pressure, and left valvular calcification with subsequent slopes in renal function. After echocardiography (performed a median of 16 days following peritoneal dialysis initiation) right ventricular systolic pressure was associated with *faster*, while declining left ventricular ejection fraction and valvular calcification were associated with *slower* declines in residual renal function. Future studies could be conducted to confirm these findings, and identify pathophysiological mechanisms.

## Introduction

Clinical guidelines and practice have led to widespread use of echocardiography in patients new to dialysis^1^. This frequently reveals abnormalities such as left atrial enlargement, left ventricular hypertrophy, impaired left ventricular function, elevated right ventricular systolic pressures, and valvular calcification that are prospectively tied to worsened survival and cardiovascular outcomes^1–5^. Links between echocardiographic findings and subsequent changes in residual renal function (RRF), particularly in patients receiving peritoneal dialysis (PD), are less clear. Most studies examining echocardiogram findings in relation to renal function were restricted to patients not yet on dialysis or to those on hemodialysis^6^. Those that included patients on PD were limited by cross-sectional or retrospective designs, imprecision of RRF measurements, short durations of follow-up, or examination of only some of the commonly reported echocardiographic parameters^7,8^.

Renal function remains very important even after PD has been initiated, and efforts to better understand factors that affect or predict its decline are warranted. Currently accepted favorable prognostic factors include use of biocompatible PD solutions as well as use of inhibitors of the renin angiotensin aldosterone system^9^. Given that cardiac and renal disease commonly overlap, early markers (as assessed by echocardiography) of cardiac disease might also predict progression of kidney disease. The Trio Trial was a multi-center trial in which incident patients in Toronto and Hong Kong were randomized to receive biocompatible or standard PD solutions for up to two years^9^. The primary outcome was slope of RRF, and all patients received transthoracic echocardiography after PD initiation according to the published study protocol. In this sub-analysis of the Trio Trial, we examined whether and how left atrial size, left ventricular hypertrophy, left ventricular ejection fraction, right ventricular systolic pressure, and left-sided valvular calcification were associated with prospective changes in RRF in patients new to peritoneal dialysis.

## Methods

### Selection criteria

All patients analyzed in the Trio Trial were eligible for this sub-analysis. Patients who did not have echocardiography within six months of PD initiation were excluded.

### Residual renal function measurements and definitions

Residual renal function - defined as the mean of 24-hour urea and creatinine clearances - was measured every 6 weeks from dialysis initiation for 2 years, or until drop-out or study closure. Only values after echocardiography were considered for this sub-analysis.

### Echocardiographic measurements, definitions, and categories

Left atrial size was defined as the anteroposterior linear dimension in the parasternal long axis view. Values were categorized as less than 30 mm, 30 to 44 mm, and 45 mm or greater. Information on left ventricular mass index was provided less consistently than commentary on the presence of left ventricular hypertrophy. Left ventricular hypertrophy was thus considered present if specifically reported, and absent if specifically reported as absent or if not mentioned. Left ventricular ejection fraction was calculated using the biplane Simpson’s method, and variously reported as a percentage (with 55% or higher considered normal) or grade (> = 55% – grade I, 45–54% - grade II, 30–44% - grade III, and <30% - grade IV). If reported as percentage it was converted to the corresponding grade. Right ventricular systolic pressure, a surrogate of systolic pulmonary artery pressure, was derived from the maximum pressure gradient difference between the right atrium and ventricle added to an estimate of right atrial pressure. It was categorized as less than 30 mmHg, 30 to 49 mmHg, and 50 mmHg or greater. Valvular calcification was assessed by number of left-sided heart valves (or respective annuli) noted to be calcified (categorized as zero, one, or two).

### Outcome measure

The primary outcome was slope in residual renal function (mean of 24-hour urea and creatinine clearances measured every 6 weeks) after baseline echocardiography.

### Statistical analyses

After data preparation in Excel (Version 15.14, Microsoft) all statistical analyses were conducted in STATA (Stata/IC 13.1, StataCorp, College Station, Texas). Descriptive statistics were reported as frequencies (%), means (standard deviations) or medians (ranges) as appropriate. Generalized mixed linear regression modeling assuming an autoregressive (1) covariate structure was used to examine associations between RRF and baseline echocardiographic parameters. The primary outcome was assessed in a mixed model that counted time on dialysis, study site, choice of PD solution and interaction term, and history of congestive heart failure, diabetes mellitus, or coronary artery disease in addition to the five echocardiographic parameters (examined individually and together) along with interaction terms as fixed-effects explanatory variables. Patient identification number was the only random-effect covariate included in the model. Two sensitivity analyses were conducted: the first assessed the effect of the timing of echocardiograms relative to PD initiation by adding the date of echocardiography as a fixed-effects covariate to the model. The second assessed whether similar results were obtained if urine volume was used as the primary outcome.

### Ethical statements

Compliance with Ethical Standards: study procedures were conducted in accordance with the Good Clinical Practice and the Declaration of Helsinki on biomedical research involving human subjects.

### Ethical approval

Written approval to conduct the Trio Trial was obtained from the ethics review boards at each of the participating hospitals - Scarborough Hospital Ethics Review Board, Credit Valley Ethics Review Board, and The Princess Margaret Hospital Ethics Review Board). The protocol was registered at controlled-trials.com/isrctn/pf/26252543 (ISRCTN26252543).

### Informed consent

All participants signed written informed consent before enrolment in the study.

## Results

Ninety-nine of 101 patients in the Trio Trial received protocolized echocardiograms within 6 months of starting PD. They were followed for a median of 685 days (range 84 to 795 days). Mean initial glomerular filtration rate was 6.8 ± 2.9 ml/min/1.73 m^2^ and overall unadjusted rate of decline was 0.154 ml/min per month (Table 1). Three patients died during the study. Echocardiograms were performed a median of 16 days after dialysis initiation (range 128 days before to 101 days after) including 22 conducted before PD initiation. The results of the echocardiograms are summarized in Table 2. Most patients had normal left ventricular ejection fractions, and for subsequent purposes this parameter was categorized as normal or reduced.

**Table 1:** Baseline characteristics of 99 patients.

Characteristic	
Age (years+/−SD)	60 ± 11
Female (number (%))	40 (40%)
Glomerular filtration rate (mL/min/1.73 m2+/−SD))	6.8 ± 2.9
Ethnicity (number (%))	
South Asian	18 (18%)
Native Canadian	2 (2%)
African Canadian	4 (4%)
Caucasian/Arab	8 (8%)
East Asian	67 (68%)
Urine volume (mL/day+/−SD)	1561 ± 640
Systolic BP (mm Hg+/−SD)	139 ± 17
Diastolic BP (mm Hg+/−SD)	74 ± 11
History of diabetes mellitus (number (%))	49 (50%)
History of coronary artery disease (number (%))	16 (16%)
History of congestive heart failure (number (%))	18 (18%)
Smoking history (number (%))	
• Never	64(65%)
• Previous	30(30%)
• Current	5(5%)
Serum calcium (mmol/L+/−SD)	2.15 ± 0.22
Serum phosphate (mmol/L+/−SD)	1.73 ± 0.43
C-Reactive Protein (mg/L+/−SD)	3 (0.2–115)

**Table 2:** Results of Echocardiograms.

Parameter and categories	
Left atrial size (mm) (median(range))	36 (26–59)
• <30	15 (16%)
• 30–45	68 (72%)
• >45	11 (12%)
Left ventricular hypertrophy present	58 (59%)
Left ventricular ejection fraction grade	
• I (normal, ejection fraction >=55%)	93 (95%)
• II (ejection fraction 45–54%)	4 (4%)
• III (ejection fraction 30–44%)	1 (1%)
• IV (ejection fraction <30%)	0 (0%)
Right ventricular systolic pressure (mmHg) (median(range))	32 (19–69)
• <30	17 (31%)
• 30–49	32 (59%)
• > = 50	5 (9%)
Number of left-sided cardiac valves (or annuli) calcified	
• none	64 (65%)
• 1	23 (23%)
• 2	12 (12%)

**Figure 1:**
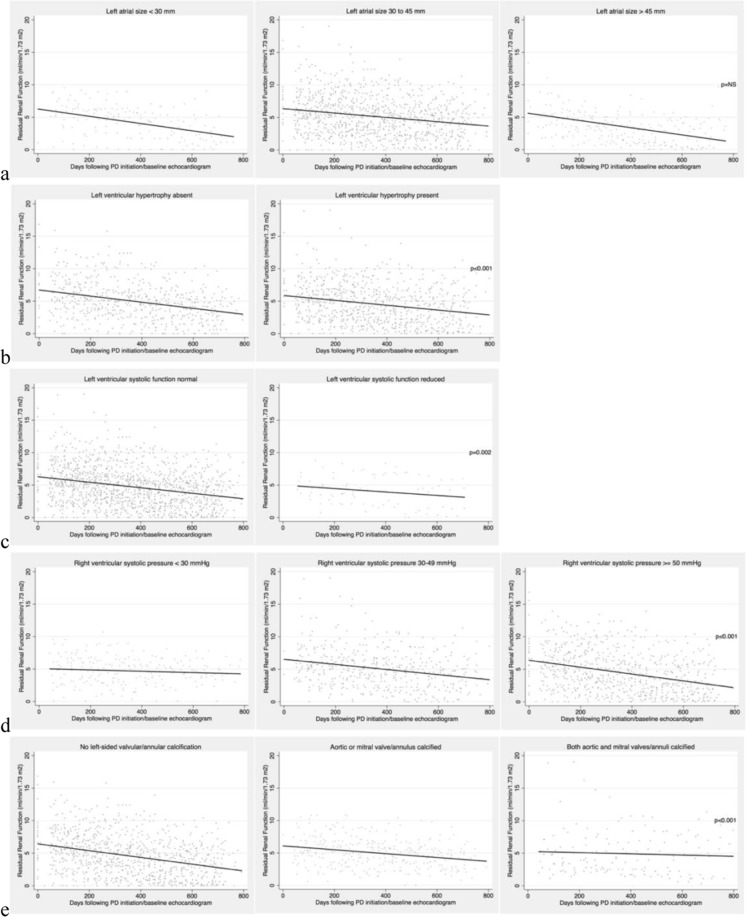
Prospective residual renal function decline stratified by echocardiograph parameter: (**a**) Left atrial size. (**b**) Left ventricular hypertrophy. (**c**) Left ventricular systolic function. (**d**) Right ventricular systolic pressure. (**e**) Left-sided valvular/annular calcification. The dots represent individual patient renal function measurements and the solid lines predicted slopes. P-values represent significance of the two-way interaction between parameter and time (taken from Table 3). PD = peritoneal dialysis.

When examined independently of the other parameters, all but left atrial size significantly associated with RRF (Table 3). Figure 1(a–e) represents actual RRF values along with fitted (predicted) rates of RRF decline according to categories of baseline echocardiographic parameters. All p-values were taken from Table 3. Collectively, left ventricular ejection fraction, right ventricular systolic pressure, and left-sided valvular (or annular) calcification remained significantly associated with RRF (Table 4).

**Table 3:** Associations* between baseline echocardiographic parameters and prospective changes in residual renal function – one parameter per model.

	Change in residual renal function (ml/min/1.73m^2^ per month) by category of parameter	95% Confidence Interval	p**
Left atrial size (mm)			NS
• <30	−0.18	−0.27 to −0.09	
• 30–45	−0.17	−0.28 to −0.06	
• >45	−0.16	−0.30 to −0.02	
Left ventricular hypertrophy			<0.001
• present	−0.15	−0.21 to −0.10	
• absent	−0.21	−0.23 to −0.18	
Left ventricular ejection fraction			0.002
• normal	−0.18	−0.20 to −0.16	
• reduced	−0.08	−0.16 to 0.00	
Right ventricular systolic pressure (mmHg)			<0.001
• <30	−0.13	−0.22 to −0.03	
• 30–49	−0.19	−0.32 to −0.06	
• > = 50	−0.25	−0.41 to −0.09	
Number of left-sided cardiac valves (or annuli) calcified			<0.001
• 0	−0.2	−0.23 to −0.18	
• 1	−0.15	−0.19 to −0.11	
• 2	−0.1	−0.16 to −0.04	

^*^One parameter (as categorized), time (since echocardiogram/PD initiation), study site, history of diabetes, congestive heart failure or coronary artery disease, peritoneal dialysis solution, and interaction (product of parameter and time) in each model. **p-values for the significance of the interaction between parameter and time.

**Table 4:** Associations* between baseline echocardiographic parameters and prospective changes in residual renal function – parameters examined collectively.

	Change in residual renal function (ml/min/1.73 m^2^ per month)	95% Confidence Interval	p**
Left atrial size (mm)			NS
• <30	−0.14	−0.28 to −0.00	
• 30–45	−0.11	−0.29 to 0.07	
• >45	−0.08	−0.30 to 0.14	
Left ventricular hypertrophy			NS
• absent	−0.17	−0.28 to −0.07	
• present	−0.15	−0.30 to −0.01	
Left ventricular ejection fraction			0.001
• normal	−0.17	−0.28 to −0.07	
• reduced	0.01	−0.20 to 0.21	
Right ventricular systolic pressure (mmHg)			<0.001
• <30	−0.25	−0.38 to −0.11	
• 30–49	−0.32	−0.49 to −0.15	
• > = 50	−0.39	−0.60 to −0.19	
Number of left-sided cardiac valves (or annuli) calcified			<0.001
• 0	−0.17	−0.28 to −0.07	
• 1	−0.12	−0.25 to 0.01	
• 2	−0.07	−0.23 to 0.09	

^*^Each parameter (as categorized), time (since echocardiogram/PD initiation), study site, history of diabetes, congestive heart failure or coronary artery disease, peritoneal dialysis solution, and interactions (products of parameters and time) in the model. **p-values are for the significance of the interaction between each parameter and time.

The results were not altered by inclusion of the date of echocardiogram relative to dialysis initiation (data not shown), and similar results were obtained if urine volume (rather than residual renal function) was the primary outcome (see Supplements: Fig. 1a–e, and Table 1).

## Discussion

In this sub-analysis of the Trio Trial, rising right ventricular systolic pressures predicted *faster*, while reduced left ventricular ejection fraction and left valvular calcification predicted *slower* subsequent rates of decline in residual renal function. These novel findings either contrast with the existing literature or have not previously been examined.

In their analysis of 2959 patients with CKD in the Chronic Renal Insufficiency Cohort, Navaneethan *et al*. found no evidence for a prospective association between pulmonary hypertension (based on echocardiogram-derived right ventricular systolic pressures) and renal events^10^. However, in defining renal events as a 50% drop in glomerular filtration rate and/or need for renal replacement therapy, the authors may have missed more subtle changes in renal function. We speculate that a rising right ventricular systolic pressure reflects progressive right ventricular diastolic dysfunction and increasing preload dependence^11^. If so, the universal and sometimes aggressive pursuit of ‘dry’ weights in dialysis-dependent patients with right ventricular diastolic dysfunction would be expected to lead to disproportionately larger drops in cardiac output. Rising right ventricular systolic pressure and declining renal function may also share a common underlying cause. For example, arterial vasoconstriction in the pulmonary and renal vascular beds (on the basis of disordered circulating endothelin-1 and nitrous oxide) has been reported in CKD^12^. Similarly, increased pulmonary and podocyte calcium-sensing receptor activity - which induces pulmonary smooth muscle cell proliferation and increased proteinuria respectively^13,14^ – presents another intriguing possibility. However, whether such activation occurs in dialysis-dependent patients has not to our knowledge been studied.

The finding that worsened left ventricular function was associated with slower rates of decline in RRF was unexpected, especially given that left ventricular function was normal in 95% and only moderately impaired in the remaining five percent of patients (Table 2). It is possible that medical therapy of congestive heart failure in patients with only moderate left ventricular dysfunction favorably impacts rates of residual renal function decline. However, we were unable to show an association between a clinical history of congestive heart failure and RRF (data not shown). Left ventricular function did not associate with RRF in another cohort of 242 new PD patients with more severe left ventricular dysfunction^15^. It is likely that the consequences of left ventricular dysfunction, as seen in other scenarios, may depend on the degree of dysfunction. For example, unlike patients with more moderate dysfunction, only those with sustained ejection fractions under 35% suffer high enough rates of arrhythmic deaths to warrant consideration of therapy with implantable cardiac defibrillators^16^.

To our knowledge, no studies have looked for a prospective association between left valvular calcification and RRF. In their cross-sectional analysis of 230 prevalent PD patients, Wang *et al*. found an inverse association between valvular calcification and RRF. However, the direction of association could not be ascertained, and the significance of this association was lost after adjusting for the calcium/phosphorus product and C reactive protein levels^7^. While it is biologically implausible that the presence of valvular calcification should delay the decline in residual renal function, these observations may be explained by the presence of variables which confound the link between valvular calcification and renal function.

Left ventricular hypertrophy associated with slower rates of decline in RRF only when examined in isolation of the other echocardiographic variables, and left atrial size did not associate with RRF in this cohort of patients. Previous studies did not collectively examine these five echocardiographic variables. For example, although Kim *et al*. showed a positive association with left atrial size, right ventricular systolic pressure was not included in their multivariate analysis that showed an association between left atrial size and RRF^8^.

### Limitations

The Trio Trial was designed and powered to ascertain changes in renal function following treatment with two different PD solutions, not after echocardiography. Furthermore, the lack of data concerning prospective residual renal function changes in relation to baseline echocardiographic parameters meant that sample size and power calculations could not be performed. The timing of the echocardiograms was also not standardized, with 22 performed before dialysis initiation, and the rest up to 101 days later. While it is unlikely that the echocardiographic parameters would have changed significantly within the time-frame allowed, this was addressed by restricting the analysis to residual renal function measured after echocardiography. This reduced the planned follow-up period for some patients, but extended the findings to those receiving echocardiograms up to 3 months post dialysis initiation. Details included in the reports were not standardized, and parameters such as diastolic function, indexed left ventricular mass, or the ability to grade left ventricular hypertrophy were not available. Finally, the impact of potential unexamined confounders (such as use of certain medications, serum phosphate and calcium and inflammatory markers) cannot be excluded, and this study is unable to conclude causality between echocardiographic findings and prospective changes in residual renal function.

### Summary

Elevated right ventricular systolic pressure associated with faster, while reduced left ventricular ejection fraction and valvular calcification associated with slower prospective rates of decline in residual renal function in this cohort of 99 incident PD patients. If confirmed, these findings might yield further insight into the prognostic value of echocardiography and the factors that affect residual renal function in patients receiving PD.

## Supplementary information

Supplement Figure 1 (a-e)
