# Supplementary material for: Associations between echocardiographic findings and prospective changes in residual renal function in patients new to peritoneal dialysis
Source: Sci Rep. 2019 Dec 5;9:18434. doi: 10.1038/s41598-019-54851-2 (PMC6895151; doi:10.1038/s41598-019-54851-2)
Supplement: Supplementary file 1 — Supplement Figure 1 (a-e) [file 41598_2019_54851_MOESM1_ESM.docx]

Associations between echocardiographic findings and prospective changes in residual renal function in patients new to peritoneal dialysis.

Sara Mahdavi^1,2^, Kibar Yared^1,3^, George Wu^3,4^, Billy Omar^1^, Dinesh Savundra^1^, Gordon Nagai^1^, Edgar Hockmann^3^, Anton Svendrovski^5^, Antonio Bellasi^6^, Paul Tam^1^, Tabo Sikaneta^1,2,3^.

^1^Department of Nephrology, The Scarborough Hospital, Toronto, Canada. ^2^Faculty of Family and Community Medicine, University of Toronto, Canada. ^3^Faculty of Medicine, University of Toronto, Canada. ^4^Department of Medicine, Trillium Health Partners, Mississauga, Canada. ^5^Private Biostatistics Consultant, Toronto, Canada. ^6^Aziende Socio Sanitarie Territoriale Papa Giovanni XXIII, Italy. Correspondence and requests for materials should be addressed to T.S. (email: tabosikaneta@yahoo.ca)

Title and legend for Supplement Figure 1 :

Supplement Figure 1: Prospective urine volume decline stratified by baseline echocardiograph parameter category:

1. Left atrial size
2. Left ventricular hypertrophy.
3. Left ventricular systolic dysfunction.
4. Right ventricular systolic pressure.
5. Left-sided valvular/annular calcification.

Legend: The dots represent individual patient daily urine volumes and the solid lines predicted slopes. P-values represent significance of the two-way interaction between parameter and time (taken from mixed models that counted time on dialysis, study site, choice of PD solution and interaction term, and history of congestive heart failure, diabetes mellitus, or coronary artery disease, and individual echocardiographic parameter and interaction term as fixed-effects explanatory variables). PD=peritoneal dialysis

1.
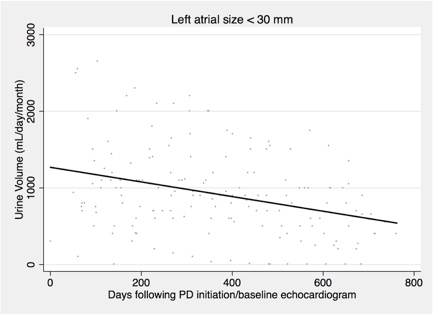

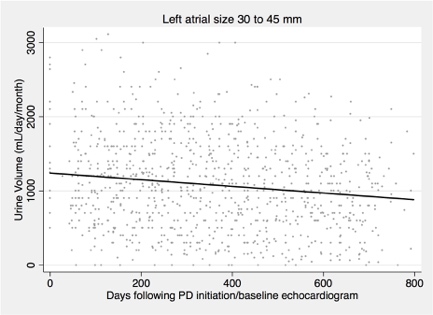

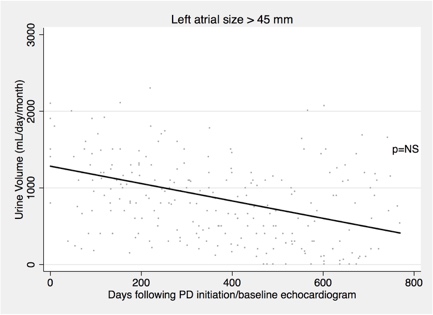

2.
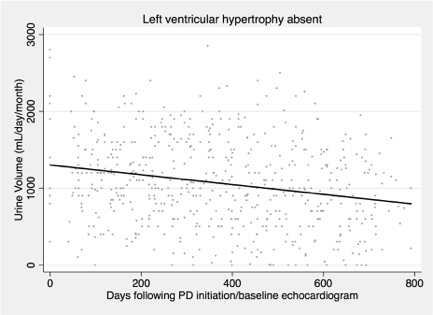

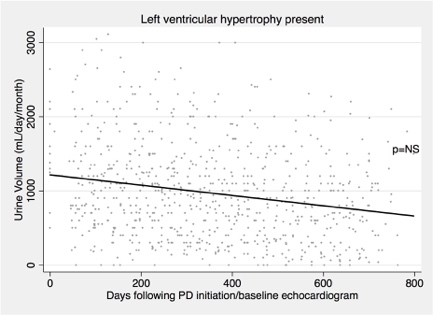

3.
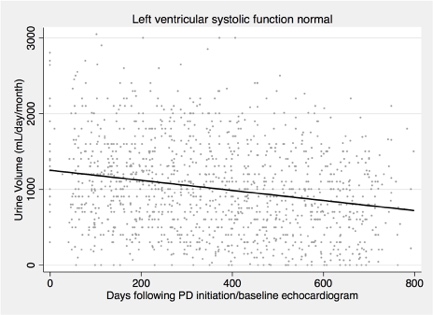

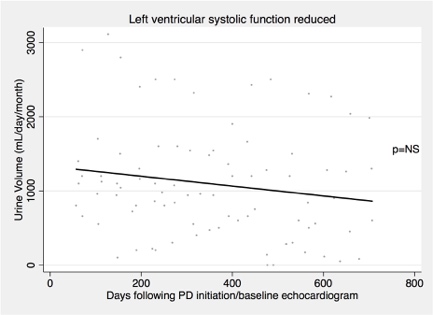

4.
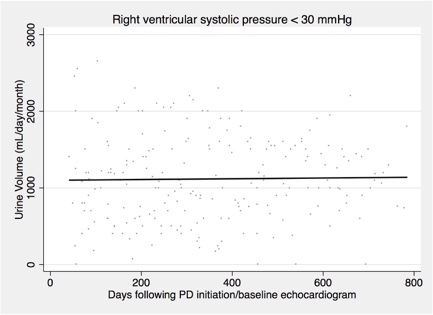

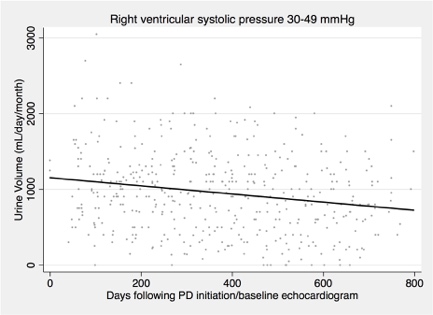

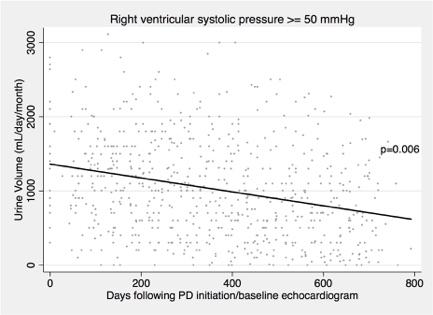

5.
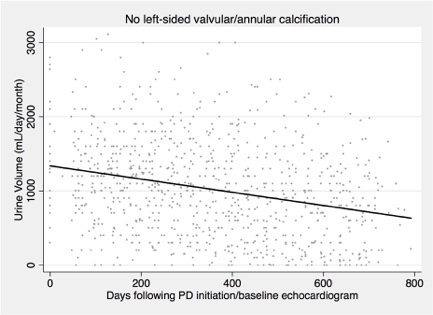

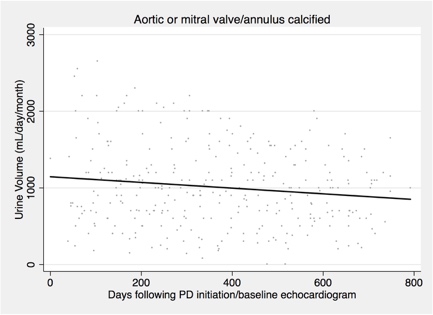

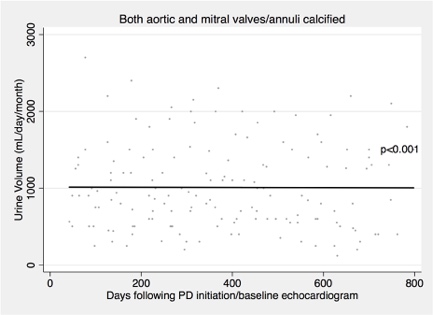


Supplement Table 1: Associations* between baseline echocardiographic parameters and prospective changes in urine volume – parameters examined collectively.

|  | **Change in urine volume**  **(ml/day/month)** | **95% Confidence Interval** | **p**** |
| --- | --- | --- | --- |
| Left atrial size (mm)   - <30 - 30-45 - >45 | -12  -12  -12 | -43 to 19  -52 to 28  -61 to 36 | NS |
| Left ventricular hypertrophy   - absent - present | -12  -15 | -35 to 11  -46 to 17 | NS |
| Left ventricular ejection fraction   - normal - reduced | -12  12 | -35 to 11  -33 to 57 | 0.035 |
| Right ventricular systolic pressure (mmHg)   - <30 - 30-49 - >=50 | -24  -36  -48 | -54 to 6  -73 to 2  -92 to -3 | 0.002 |
| Number of left-sided cardiac valves (or annuli) calcified   - 0 - 1 - 2 | -12  -4  -3 | -35 to 11  -33 to 24  -31 to 38 | 0.013 |

*Each parameter (as categorized), time (since echocardiogram/PD initiation), study site, history of diabetes, congestive heart failure or coronary artery disease, peritoneal dialysis solution, and interactions (products of parameters and time) in the model. **p-values are for the significance of the interaction between each parameter and time.
